# Intangible features extraction in the processing of abstract concepts: Evidence from picture-word priming

**DOI:** 10.1371/journal.pone.0251448

**Published:** 2021-05-11

**Authors:** Dounia Lakhzoum, Marie Izaute, Ludovic Ferrand

**Affiliations:** Université Clermont Auvergne, CNRS, LAPSCO, Clermont-Ferrand, France; CNRS - Université d’Aix-Marseille, FRANCE

## Abstract

Over the last decade, hypotheses ranging from linguistic symbol processing to embodiment have been formulated to account for the content and mechanisms responsible for the representation of abstract concepts. Results of recent studies have suggested that abstract concepts, just like concrete ones, can benefit from knowledge of real-world situational context, but that they can also be processed based on abstract pictures devoid of such situational features. This paper presents two semantic priming experiments to explore such mechanisms further. The first experiment replicates Kuipers, Jones, and Thierry (2018) in a cross-linguistic setting which shows that abstract concepts can be processed from abstract pictures devoid of tangible features. In the second experiment, we studied extraction mechanisms that come into play when participants are presented with abstract and concrete pictures that provide situational information to illustrate target abstract concepts. We expected this facilitatory effect to be limited to concrete picture primes. Our data analysed with both Bayesian and Frequentist tests showed however that even when presented with tangible situational information, the extraction of features still occurred for abstract pictures. We discuss the implications of this with respect to future avenues for studying the processing of abstract concepts.

## Introduction

Abstract concepts have long been a riddle for the brain and cognitive sciences. The past decade saw a greatly enhanced research effort as regards abstract concepts representation (e.g., [1]; see [2] for a review). In spite of such efforts, challenging questions remain as to how abstract concepts convey meaning and the type of information in which their representation is encoded.

### Dimensions of abstract concepts

With abstract concepts, there is no one-to-one matching between the concept–word and a single referent. Usually they refer to complex situations involving multiple objects and entities [3–5]. Until recently, their definition was formulated primarily in opposition to concrete concepts, therefore constraining the scope of the debate because the dichotomy between abstract and concrete concepts is not clear-cut [6]. For a long time, the most commonly used dimension was imageability, with concrete concepts referring to highly imageable entities that can be perceived through the senses and abstract concepts having low imageability. This dimension is the direct consequence of Paivio’s Dual Coding Theory (DCT, [7]) according to which concrete concepts trigger processing based on two informational systems, one visual, the other verbal, whereas abstract concepts are processed only in the verbal system. More specifically, imageability is the ability of “words [to] arouse sensory experience” (Paivio et al., 1968, p.4; see also [8]). In that sense, it is implicitly equated with abstractness, so much so, in fact, that abstract concepts stimuli were often chosen among words with low imageability ratings.

A closely related dimension used to distinguish abstract concepts comes from the Context Availability Theory (CAT, [9]) according to which concrete concepts refer to a set number of contexts whereas abstract concepts are loosely connected to a variety of contexts. The argument here is that the availability of a strong network of highly defined features provides concrete concepts with a processing advantage over abstract concepts. This distinction perpetuates the inaccurate view that abstract concepts are poor in features. It has been somewhat invalidated, however, by recent evidence showing that although abstractness is highly correlated with imageability, the two dimensions are not equivalent. Kousta, Vigliocco, Vinson, Andrews, and Del Campo (2011) found a processing advantage in favour of abstract concepts once the imageability and context variables were controlled, a finding which intensified the perceived need to uncover more dimensions to define abstract concepts [10]. To that end, Villani et al., (2019) introduced a novel database of 15 dimensions clustered around sensorimotor, linguistic, inner, social states and hand and mouth effectors [5]. The database offers researchers the ability to select abstract stimuli along more defined dimensions and allows for better-controlled experiments.

The study of abstract concepts is benefiting from the greater emphasis placed on their variety, dimensions and richness. Not only has this intensified interest been concerned with defining their dimensional content, it has also produced theoretical accounts to explain how they are processed. Several theories on a spectrum ranging from amodal to grounded assumptions have been formulated to answer questions about processing mechanisms for abstract concepts.

#### Theoretical accounts in the study of abstract concepts

At the amodal end of the spectrum, traditional accounts of semantics use symbolic representations to define the meaning of concepts [11–14]. In recent years, this account has been integrated in models of distributional semantics able to extract meaning from statistical distribution across corpora (see [15] for a review of these models). Given that word co-occurrences can be computed for concrete and abstract concepts alike, this account finds no issue with explaining the representation of abstract concepts. However, use of amodal linguistic symbols as a proxy for representing meaning has been under fire for its lack of grounding in modality-specific brain areas [16–20].

At the grounded end of the spectrum, the meaning of concepts is derived from perceptual and motor states [21–23]. For instance, Pulvermüller, Shtyrov, and Ilmoniemi (2005) used MEG techniques to show that brain areas responsible for motor actions of the face or leg are activated when action words such as *lick* or *kick* are processed [24]. Such evidence is challenged, however, when faced with abstract concepts such as *truth* or *freedom*, as they do not refer directly to physical features of the world. That explains why the embodied account had to be extended to include hypotheses for the grounding of abstract concepts.

Several studies have investigated the representation of abstract concepts through feature-listing paradigms and have demonstrated that abstract concepts also benefit from some form of modality-specific representation. For example, abstract concepts activate social and introspective aspects of situations [25,26], emotional features [27], event information and thematic roles [28]. More generally, these studies unveiled representation mechanisms for abstract concepts that place greater emphasis on the context in which they are used (e.g., [29–31]). For instance, Wilson-Mendenhall, Martin, Simmons and Barsalou (2013) performed a task requiring deep conceptual processing of abstract concepts such as *convince* [32]. They observed neural activation patterns of non-linguistic brain areas associated with mentalizing and social cognition. In a more recent study, Harpainter et al. (2020) studied brain activation patterns for visual and motor abstract concepts such as *beauty* and *fight* [33]. In line with the grounding of representation framework, their results suggest some categories of abstract concepts benefit from a similar grounding mechanism to concrete concepts. Middle-ground theories, however, consider that both amodal and grounded content contribute to the representation of abstract concepts. For instance, Dove’s representation pluralism ([34–36] see also [2] and [37] for a review) represents just such a hybrid approach, according to which abstract concepts activate both linguistic and modality-specific features.

#### Picture priming paradigms in the study of abstract concepts

The representation of abstract concepts in situations and contexts has been further investigated in recent years [25,29,30]. More recently, McRae, Nedjadrasul, Pau, Pui-Hei Lo and King (2018) presented abstract target words primed by pictures depicting scenes either related or unrelated to the target words [38]. For example, the concept *discipline* was primed by the picture of students lined up. When target words were preceded by related pictures, lexical decision latencies were shorter than when unrelated pictures were shown. Such results support the assumption of extraction of situational features in abstract concept processing.

In another recent study, Kuipers, Jones and Thierry (2018) used a similar priming paradigm, but with abstract picture primes, which lack tangible situational features [39]. The results showed shorter manual latencies and a smaller N400 amplitude in the EEG data for related abstract picture-word pairs than unrelated ones. The authors inferred that the abstract pictures conveyed the same meaning as the abstract words. The fact that abstract images devoid of situational context elicited an activation pattern similar to the abstract concepts they primed suggests the need for further investigation of the mechanisms involved in abstract concept processing.

#### The present study

After norming the abstract picture-word pairs from Kuipers et al. (2018) with French participants, we replicated their study in French in a first experiment to make sure their findings remained valid in a cross-linguistic setting. In a second experiment, we expanded on their study by comparing abstract picture primes to concrete ones within the same experiment [39]. For the first experiment, we expected to find the same facilitatory effect as in English, with shorter manual latencies for related abstract picture-word pairs than for unrelated ones. In a second experiment, we compared concrete picture primes allowing for situational grounding to abstract picture primes composed of intangible features. For this second experiment, in which concrete and abstract picture primes were presented together we also expected shorter latencies for related picture-word pairs than for unrelated ones in the concrete picture condition. However, and in line with the previously discussed role of situational features in the representation of abstract concepts, we expected this facilitatory effect to be limited to concrete picture primes, insofar as they allow for a more tangible form of situational or contextual information, and to disappear in respect of abstract picture primes (see [38]). Tangible features, as opposed to intangible ones, should allow for easier processing [40]. We expected that when presented with both types of features the participants would develop a strategy by allocating more resources to extracting tangible features, to the detriment of intangible ones.

## Experiment 1

### Materials and methods

#### Participants

The same number of participants were tested in Experiment 1 as were tested by Kuipers et al. (2018) [39]. Twenty native French speakers from Université Clermont Auvergne, France, took part in this experiment (4 males; M_age_ = 24, SD = 3). All participants were right-handed with corrected-to-normal vision. They all gave their informed written consent before taking part in the study. They were rewarded for their participation with a 10€ gift card. The study was approved by the local ethics committee (Comité d’éthique de la Recherche IRB-UCA).

#### Materials

**Fig 1:**
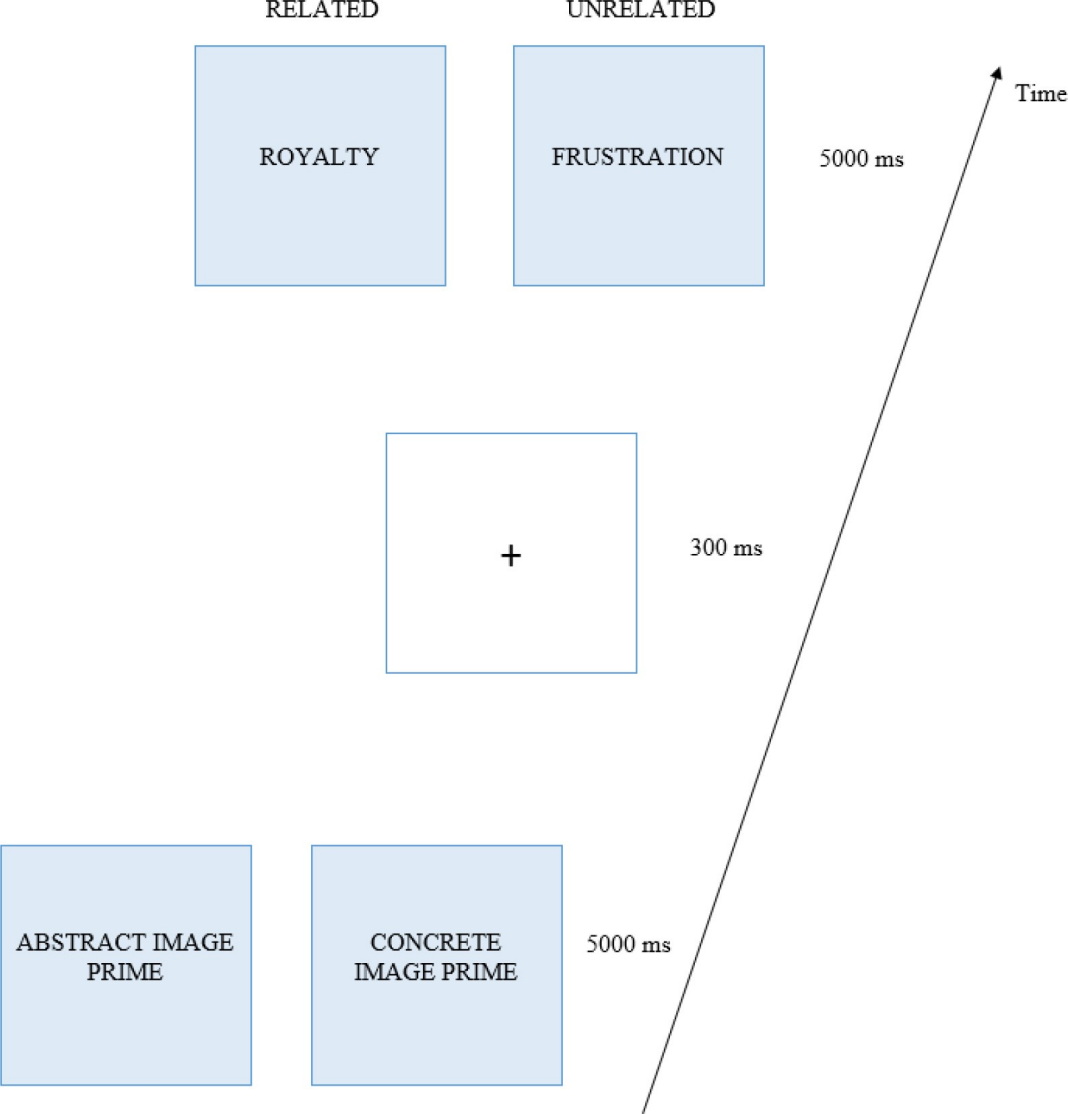
Trial procedure for the concept royalty (“royauté” in French). Note. For this concept, each participant was exposed to either the abstract or the concrete image prime followed by the related or unrelated target word. The trial procedure for Experiment 1 consisted of only the abstract images paired with related or unrelated words, whereas Experiment 2 also had concrete images in addition to the abstract pictures. The abstract image stimulus is a fractal created by Sven Geier (http://www.sgeier.net/fractals/fractals/07/The%20Road%20Ahead.jpg) and the concrete image stimulus is a coronation picture credited to the National Film Board of Canada. Photothèque. Library and Archives Canada, PA-196667; CC BY 2.0; https://tinyurl.com/pxfvv8p7).

The material used in Experiment 1 was crafted on the basis of the norming study described hereafter. For Experiment 1, the purpose was to create a set of semantically related and unrelated picture-word combinations based on the stimuli used in [39]. We obtained the abstract images and words collected for their study by contacting the authors. The materials consisted of 100 abstract images, each paired with one related and one unrelated abstract word. We translated the words into French and submitted the picture-word combinations to a group of 166 French participants (56 men, M_age_ = 21.7, SD = 4.6) using Qualtrics (2020) in a preliminary study. As in Kuipers et al. (2018), participants were asked, “How strongly does the word below match the above picture?” (on a scale of 0 to 10) [39]. We kept only the 71 picture-word combinations that elicited the highest scores for the related conditions, and those that elicited the lowest scores for the unrelated conditions (M_Related_ = 6.5, SD = 1.0; M_Non_Related_ = 2.2, SD = 1.1; t(70) = 21.6, p < 0.001, d = 2.6, 95CI [2.1–3.0], see Fig 1 for an example of combination).

#### Procedure

Experiment 1 used exactly the same procedure as Kuipers et al. (2018), except that we recorded only reaction times, and not EEG. We presented abstract image-word combinations that were either semantically related or unrelated (see Fig 1 for example stimuli) [39]. Both the related and unrelated modalities were presented in a within-subjects design. Stimuli were presented using E-prime software v2.1 on a 15-inch Dell PC colour monitor with a refresh rate of 60 ms and connected to an AZERTY keyboard. Participants were seated at a distance of 50 cm from the monitor. Each trial began with a fixation cross, displayed in the middle of the screen for 300ms followed by an abstract picture that remained on the screen for 5000ms. The fixation cross was then replaced with an abstract target word, which stayed on the screen until the participant answered, or until 5000ms had elapsed. The participant’s task was to indicate, using the D and K keys on the AZERTY keyboard, whether the target word was semantically related or unrelated to the previous picture. Response latencies were recorded, as well as accuracy. The experiment took approximately 20 minutes to complete.

### Results

#### Reaction times

**Fig 2:**
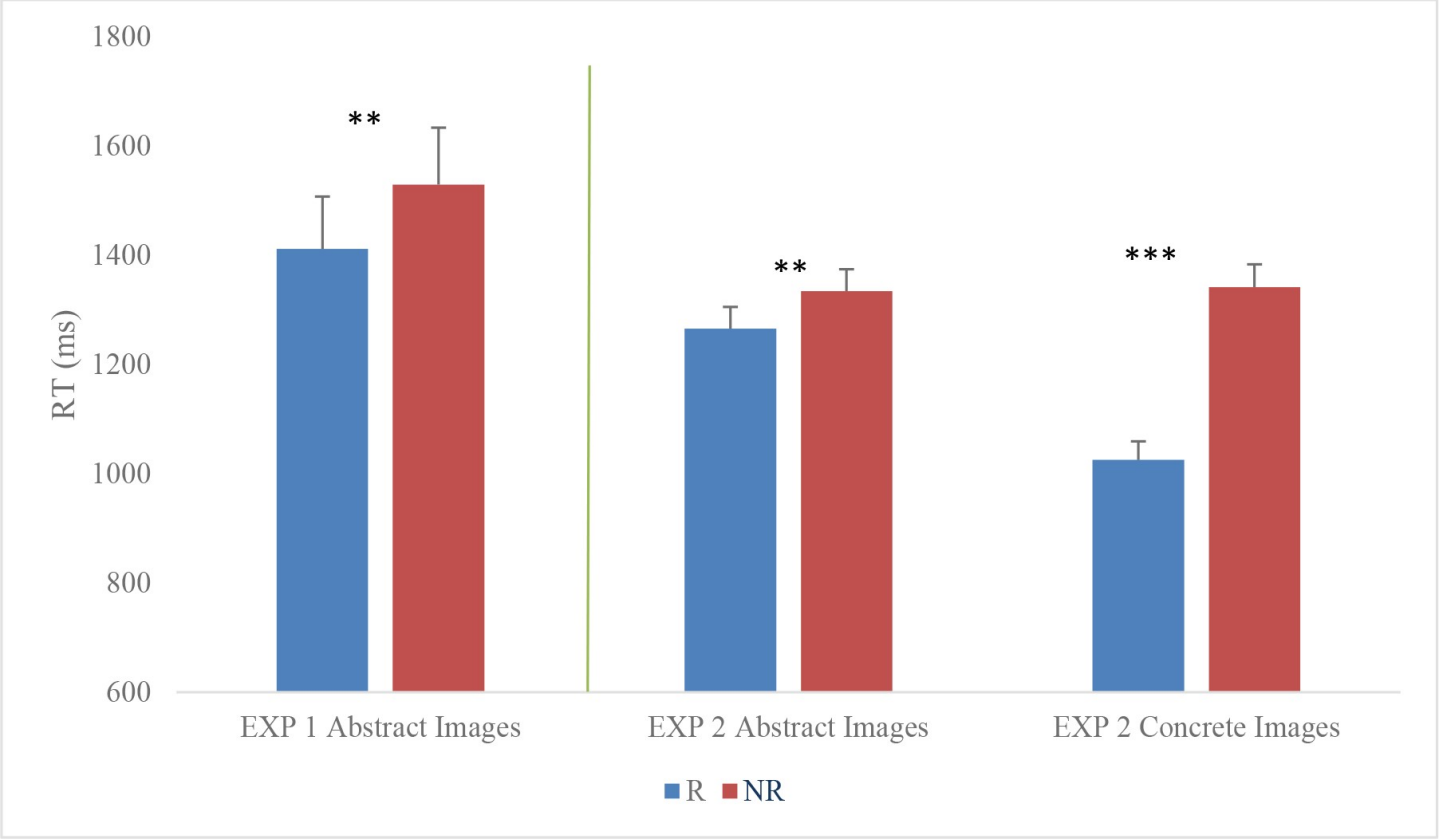
Mean correct latencies (in ms with standard error bars) for type of image prime and type of target word. (R = related pairs; NR = non-related pairs; **p = 0.01; ***p < 0.001) in both Experiments 1 and 2.

Latencies > 3 SDs above or below each participant’s mean latencies for each condition were excluded from the analyses (less than 2% of the data). Mean correct latencies were analysed by means of a one-sided paired-samples t-test testing the same directional hypothesis as Kuipers et al., (2018). We conducted analyses within a Bayesian framework relying on prior specification of theoretical knowledge which includes effect sizes and the direction of hypotheses [33], see also [41]. The analysis revealed an effect of Target Word Type with shorter latencies for the related pairs than the unrelated ones (M_Related_ = 1411 ms; SD = 429; M_Non_Related_ = 1529 ms; SD = 464; Mean Difference = 118 ms; 95CI [21, 214]; t(19) = 2.55, p = 0.01, Cohen’s d = 0.57, see Fig 2). We conducted a Bayesian paired-samples t-test using JASP [42]. For the informed prior we used Oosterwijk’s recommendation (t-distribution with location 0.350, scale 0.102, and 3 degrees of freedom), because it is considered a good prior for small-to-medium effect sizes (see [43]). This analysis showed a Bayes Factor of 10.93 (median = 0.39; 95CI [0.19–0.67]). This means the results are about 11 times more likely under the alternative hypothesis compared to the null. Taken together, the Bayesian analyses show substantial to strong support for the finding that abstract images can elicit a semantic priming effect [44].

Yet, this effect is somewhat surprising and rather counter-intuitive with respect to the role of situational grounding in concept representation. We therefore conducted supplementary analyses in a Bayesian replication framework to test for the robustness of such an effect. To that end, we applied a Replication Test in Rstudio [45] (ReplicationBF package [46]) which can be used to assess the success or failure of a replication attempt by testing whether the effect identified in previous studies is present or absent in the replication attempt [46], whether the effect size in the replication is equal to that found in the original study [47], and whether the effect is present when the data are pooled in a meta-analysis [48]. The replication test relies on sample sizes and t-values and requires no specification of a prior distribution, thus escaping the influence of a researcher’s choice of priors, but it uses the posterior distribution of the original study as a prior for the replication attempt ([49,50] but see [51]). The results of the replication tests are reported in the supplementary analyses. For the equality-of-effect size Bayes factor, support for the null hypothesis, according to which there is no difference between effect sizes, is indicative of a successful replication. The tests provided substantial support for the equality of effect sizes (Equality B01 ≈ 5; see Table A.1 in S1 Appendix), with the fixed-effect meta-analysis Bayes factor test showing strong support for the presence of an overall effect (Meta B10 ≈ 30). Finally, the new Bayes factor for replication shown in Figure A.1 in S1 Appendix indicates that the results are about 12.5 times more likely under the proponent’s hypothesis that the effect is consistent with the one found in the original study as opposed to the sceptic’s hypothesis that the effect is spurious [46].

#### Errors

A paired-samples t-test showed no effect of Target Word Types for the errors (t(19) = 0.14; p = 0.90 *ns*).

### Discussion

The aim of Experiment 1 was first to extend the replicability of Kuipers et al. (2018) to another language before considering expanding it in a follow-up experiment [39]. As in the study by Kuipers and colleagues, we obtained shorter latencies for related combinations than for unrelated ones. They interpreted their results in terms of meaning processing and concluded that it was possible that abstract pictures can convey the same meaning as lexical abstract concepts. Given the counter-intuitive results obtained by Kuipers et al. (2018), especially given the important role of situational information, we decided to conduct Bayesian analyses [46], the results of which provided strong evidence in favour of Kuipers and colleagues’ alternative hypothesis, according to which abstract pictures can convey the same meaning as abstract concepts. Furthermore, we conducted a supplementary replication test to ensure our attempt at replication was successful. We demonstrated therefore that the effect of picture-word priming using abstract pictures could be replicated in a cross-linguistic setting using different abstract images. Despite our analyses confirming the evidence uncovered by Kuipers and colleagues we do not rule out other possible interpretations (see General Discussion). Having confirmed the replicability of a study using such stimuli, we are now keen to expand Experiment 1 in a paradigm that would allow abstract concepts to be extracted from situational pictures. Experiment 2 aimed to implement such a paradigm. To that end, we kept the abstract priming pictures from Experiment 1 and added a condition for which participants were presented with concrete priming pictures that provided situational information.

## Experiment 2

### Materials and methods

#### Participants

An independent group of 90 students from Université Clermont Auvergne who met the same criteria as for Experiment 1 took part in Experiment 2 (18 males; M_age_ = 20.2; SD = 2.86). They all gave their informed written consent before taking part in the study. The study was approved by the local ethics committee (Comité d’éthique de la Recherche IRB-UCA).

#### Materials

The materials used for Experiment 2 were the result of a second norming study. The purpose of the norming study for Experiment 2 was to find corresponding concrete images for each picture-word combination in Experiment 1. To that end, we searched online databases for images likely to elicit the same meaning as the abstract words. For each word, we selected two potential images which we then submitted to an independent group of 146 French participants (38 men, M_age_ = 22.4, SD = 5.6) using Qualtrics. Participants were asked, “How strongly does the word below match the above picture?” (on a scale of 0 to 10). Picture-word combinations that obtained a low score (< 5 on a scale of 0 to 10) for both image options were discarded. The remaining 56 stimuli combinations (abstract/concrete image-related/unrelated word) were used in Experiment 2 (see Fig 1 for an example of stimuli).

#### Procedure

We used a similar procedure as for Experiment 1, but expanded it with 2 within-subjects variables: Priming Image Type (abstract vs. concrete) and Target Word Type (related vs. unrelated). The stimuli were divided in a Latin square with 4 modalities: abstract prime images, concrete prime images, semantically-related target words and unrelated target words. Following this Latin-square design, the pictures were divided into two lists so that each picture presented in the related condition to participant 1 was also presented to participant 2 in the unrelated condition. Participants were exposed to all variables but saw only one of the 4 possible types of picture-word combination for each stimulus (see Fig 1 for an illustration of the trial procedure with an example of stimuli).

### Results

#### Reaction times

Latencies > 3 SDs above or below each participant’s mean latencies for each condition were excluded from the analyses (i.e. ~ 2% of the total data). Data from 3 participants were discarded based on z-scores that showed very slow RTs in all conditions (z-scores > 2.99). The following analyses are based on the data from 87 participants.

Mean correct latencies are presented in Fig 2. Mean correct latencies were analysed with a 2(Priming Image Type = Concrete vs. Abstract) * 2(Target Word Type = Related vs. Unrelated) repeated measures ANOVA.

This analysis revealed a main effect of Priming Image Type with shorter latencies for concrete images compared to abstract images (respectively, M_Concrete_Image_ = 1183 ms; SD = 357; M_Abstract_Image_ = 1338 ms; SD = 383; Mean Difference = 116 ms; 95CI [80, 152]; F(1, 86) = 40.65, p < 0.001, ɳ_p_^2^ = 0.32). The results also showed a main effect of Target Word Type with shorter latencies for the related targets compared to unrelated targets (M_Related_ = 1144 ms; SD = 346; M_Non_Related_ = 1300 ms; SD = 372; Mean Difference = 193 ms; CI [145, 241]; F(1, 86) = 63.74, p < 0.001, ɳ_p_^2^ = 0.43). The results showed an interaction effect between Priming Image Type and the Target Word Type (F(1, 86) = 70.48, p < 0.001, ɳ_p_^2^ = 0.45). Contrast analyses were conducted to investigate this interaction further. There was a significant effect of word type within each level of image type, with shorter latencies for the related targets compared to unrelated targets for the abstract images (M_Abstract_Related_ = 1265; SE = 40; 95CI [1185, 1344]; M_Abstract_NonRelated_ = 1334; SE = 40; CI [1255, 1414]; t(86) = 2.33; p = 0.011; *d* = 0.3; CI [0.04, 0.5]) and concrete images (M_Concrete_Related_ = 1025; SE = 34; CI [957, 1093]; M_Concrete_NonRelated_ = 1341; SE = 42; CI [1258, 1426]; t(86) = 12.00; p < 0.001; *d* = 1.3; CI [0.99, 1.56], see Fig 2). The semantic facilitatory effect was therefore present for both types of prime stimuli.

A Bayesian repeated measures ANOVA compared four models to the null model. We kept the default JAPS prior for fixed effects (r scale prior width = 0.5; [47,48]). Based on Bayes Factors, the model including only the types of priming images was 5770 times more likely than the null model. There is therefore strong evidence for the type of prime model. There was also strong evidence for the model including only the type of word target (BF = 1.902x10^13^), for the model including both main effects (BF = 2.990x10^18^), and for the interaction model (BF = 2.551x10^25^). Finally, we compared the main effects model to the interaction model (2.551x10^25^/2.990x10^18^ = 8.533 x10^6^; based on the Baws Factor suggested by Mathôt, 2017 [52]) and obtained strong evidence for the interaction model.

We used a Bayesian paired-samples t-test to explore this interaction, with an informed prior following Oosterwijk’s recommendation. The analysis showed a Bayes Factor of 7.67 (median = 0.30; CI [0.13, 0.45]) in favour of the alternative hypothesis for the difference between related and unrelated target words in the abstract picture primes condition, and a Bayes Factor of 4.42x10^15^ (median = 1.19; CI [0.89, 1.48]) in favour of the alternative hypothesis for the difference between related and unrelated target words in the concrete picture primes. These Bayesian analyses concur with the ones obtained from traditional paired-samples t-test and confirm substantial evidence for the effect of the abstract picture primes and extreme evidence for the concrete ones.

#### Errors

Given the difficulty of the task, especially concerning the abstract image condition, we expected larger rates of errors overall, but more specifically for the abstract image primes.

Indeed, a repeated measures ANOVA revealed a main effect of Priming Image Type with a higher percentage of errors for the abstract images compared to the concrete images (M_Abstract_Image_ = 29%; SD = 16.5; M_Concrete_Image_ = 12%; SD = 10; F(1,87) = 189.87, p < 0.001, ɳ_p_^2^ = 0.69). As for Experiment 1, the results showed no main effect of Target Word Type.

### Discussion

Experiment 2 expanded on Experiment 1 by comparing abstract to concrete pictures priming abstract lexical concepts. We opted for a semantic, as opposed to a lexical decision task given that Recchia and Jones (2012) suggested the latter did not yield deep semantic processing and therefore accounted for the discrepancies between results in the abstract concept processing literature [53]. Recognising this might be a concern, however, we checked for such surface features based on analyses of emotional valence in an additional study. We presented the pictures to an independent pool of 49 participants online and asked them to rate the emotional valence of the pictures on a scale of 0–10. We then used this valence variable in a *post-hoc* analysis and found it failed to explain the variance in latencies for semantic priming (based on a repeated measures ANOVA (F(2, 48) = 1.76; p = .19 ns)). Based on this analysis we can rule out a shallow picture-word association based only on surface physical features.

This facilitatory effect was significantly stronger for concrete pictures than for abstract ones. However, it was weaker in relation to Experiment 1, although it did not disappear for abstract pictures.

This result expands on those obtained by Kuipers et al. (2018), showing that abstract concepts can be processed on the basis of abstract and concrete pictures alike and more broadly from tangible and intangible features. The Bayesian models provide decisive evidence in favour of the alternative hypothesis of semantic priming. The error rates obtained were quite high compared to traditional effects in the semantic priming literature. We were not surprised by these rates, however, as the task was more difficult in comparison to traditional priming studies. We asked Kuipers et al. (2018) for the error rates for their study (27% error rates for the related and 31% for the unrelated items), which confirmed our intuition that such stimuli would elicit higher error rates compared to more traditional semantic priming studies.

## General discussion

The aim of the present studies was to investigate conceptual processing mechanisms based on picture-word combinations in terms of the role of situational features compared to intangible abstract features.

In the first experiment and in line with Kuipers and colleagues, we investigated conceptual processing mechanisms in relation to abstract pictures. The results showed participants were able to process concepts from abstract pictures devoid of tangible features (therefore replicating Kuipers et al., 2018 results and extending them to the French language). To the best of our knowledge, Kuipers et al. (2018) were the first to provide evidence that abstract pictures can activate the meaning of abstract concepts. The data of Experiment 1 which was analysed using a Bayesian replication framework, corroborated their findings. This fact alone broadens the scope of conceptual representation beyond the usual debate of linguistic vs. situational features (see for e.g., [14,25,34]). It also goes to show that abstract concepts are even richer and activate features beyond their linguistic or situational components [53].

In a second experiment, we gave participants an opportunity to extract situational features (see also [38]). Results showed a stronger facilitatory effect for concrete picture-words pairs compared to abstract ones. Participants relied more heavily on the priming pictures with distinguishable features. Our results support previous findings according to which some types of abstract concepts are grounded in situations and events [25,32,40].

From the evidence from the two experiments, it appears that is possible to represent abstract concepts based on abstract and intangible pictures as well as on concrete and tangible ones. McRae et al. (2018) showed that in a picture-priming paradigm similar to our “concrete images” condition, participants could process the meaning of abstract concepts derived from pictures depicting scenes. Kuipers et al. (2018) showed how this processing could also be derived from abstract pictures that differ from the stimuli used by McRae and colleagues because they are devoid of tangible features. In the present study, and based on the assumption that it is costlier to extract intangible features when tangible ones are available, we showed that extraction mechanisms still occurred in the case of abstract pictures.

The immediate interpretation of these results makes reference to how Kuipers and colleagues and McRae and colleagues interpreted meaning processing based on tangible and intangible features. This initial interpretation infers that there is a semantic level of processing and implies a featural view of semantic representation according to which concepts can be broken up into a set of defining features that represent their meaning (see [54] for an account of the featural view). If confirmed, this assumption would mean such features can be other than lexical or situational, namely also abstract and intangible.

For instance, Bolognesi and Vernillo (2019) proposed ‘Abstraction by Metonymy’ as a novel grounding mechanism for abstract concepts in the pictorial mode, where they use verbo-pictorial metaphors to investigate people’s ability to illustrate abstract concepts [55]. According to their hypothesis, this abstraction process allows for an inferential mechanism that moves from concretely depicted entities to more abstract ones. In the present studies, the reverse mechanism seems to have occurred. It might be that in the abstract image condition, participants extracted intangible features and inferred a more tangible representation of the concept. Therefore, these results could be construed as complementing the Abstraction by Metonymy.

In conclusion, by directly comparing the two types of features for representation, we were able to show that abstract concepts can be processed based on situational features and abstract ones. For us, these conclusions signal the need to explore further the mechanisms of concept representation and abstraction.

## Supporting information

S1 Appendix Appendices: Supplementary analyses. (DOCX)

## References

[1] Lakhzoum D, Izaute M, Ferrand L. Semantic similarity and associated abstractness norms for 630 French word pairs. [cited 11 Oct 2020]. 10.3758/s13428-020-01488-z 33006067

[2] Borghi AM, Binkofski F, Castelfranchi C, Cimatti F, Scorolli C, Tummolini L. The challenge of abstract concepts. Psychol Bull. 2017;143: 263–292. 10.1037/bul0000089 28095000

[3] Barsalou LW. Abstraction in perceptual symbol systems. Philosophical Transactions of the Royal Society B: Biological Sciences. Royal Society; 2003. pp. 1177–1187. 10.1098/rstb.2003.1319 PMC1693222 12903648

[4] Pulvermüller F. Neural reuse of action perception circuits for language, concepts and communication. Progress in Neurobiology. Elsevier Ltd; 2018. pp. 1–44. 10.1016/j.pneurobio.2017.07.001 28734837

[5] Villani C, Lugli L, Liuzza MT, Borghi AM. Varieties of abstract concepts and their multiple dimensions. Lang Cogn. 2019;11: 403–430. 10.1017/langcog.2019.23

[6] Della Rosa PA, Catricalà E, Vigliocco G, Cappa SF. Beyond the abstract-concrete dichotomy: Mode of acquisition, concreteness, imageability, familiarity, age of acquisition, context availability, and abstractness norms for a set of 417 Italian words. Behav Res Methods. 2010;42: 1042–1048. 10.3758/BRM.42.4.1042 21139171

[7] Paivio A, Yuille JC, Madigan SA. Concreteness, imagery, and meaningfulness values for 925 nouns. J Exp Psychol. 1968;76: 1–25. 10.1037/h0025327 5672258

[8] Dellantonio S, Job R, Mulatti C. Imageability: Now you see it again (albeit in a different form). Frontiers in Psychology. Frontiers Media SA; 2014. p. 991. 10.3389/fpsyg.2014.00279 24765083 PMC3982064

[9] Schwanenflugel PJ, Harnishfeger KK, Stowe RW. Context availability and lexical decisions for abstract and concrete words. J Mem Lang. 1988;27: 499–520. 10.1016/0749-596X(88)90022-8

[10] Kousta ST, Vigliocco G, Vinson DP, Andrews M, Del Campo E. The Representation of Abstract Words: Why Emotion Matters. J Exp Psychol Gen. 2011;140: 14–34. 10.1037/a0021446 21171803

[11] Collins AM, Loftus EF. A spreading-activation theory of semantic processing. Psychol Rev. 1975;82: 407–428. 10.1037/0033-295X.82.6.407

[12] Pylyshyn ZW. Cognitive representation and the process-architecture distinction:Cognitive representation and the process-architecture distinction. Behavioral and Brain Sciences. Cambridge University Press; 1980. pp. 154–169. 10.1017/S0140525X00002302

[13] Fodor JA, M.I.T. Press. The mind doesn’t work that way: the scope and limits of computational psychology. MIT Press; 2001.

[14] Mahon BZ, Caramazza A. Concepts and Categories: A Cognitive Neuropsychological Perspective. Annu Rev Psychol. 2009;60: 27–51. 10.1146/annurev.psych.60.110707.163532 18767921 PMC2908258

[15] Andrews M, Frank S, Vigliocco G. Reconciling Embodied and Distributional Accounts of Meaning in Language. Top Cogn Sci. 2014;6: 359–370. 10.1111/tops.12096 24935903

[16] Gallese V, Lakoff G. The brain’s concepts: The role of the sensory-motor system in conceptual knowledge. Cognitive Neuropsychology. 2005. pp. 455–479. 10.1080/02643290442000310 21038261

[17] Pulvermüller F. Brain mechanisms linking language and action. Nature Reviews Neuroscience. Nature Publishing Group; 2005. pp. 576–582. 10.1038/nrn1706 15959465

[18] Barsalou LW. Grounded Cognition. Annu Rev Psychol. 2008;59: 617–645. 10.1146/annurev.psych.59.103006.093639 17705682

[19] Borghi AM, Cimatti F. Embodied cognition and beyond: Acting and sensing the body. Neuropsychologia. 2010;48: 763–773. 10.1016/j.neuropsychologia.2009.10.029 19913041

[20] Meteyard L, Cuadrado SR, Bahrami B, Vigliocco G. Coming of age: A review of embodiment and the neuroscience of semantics. Cortex. 2012;48: 788–804. 10.1016/j.cortex.2010.11.002 21163473

[21] Barsalou LW. Perceptual symbol systems. Behavioral and Brain Sciences. 1999. pp. 577–609. 10.1017/s0140525x99002149 11301525

[22] Glenberg AM. Mental models, space, and embodied cognition. Creative thought: An investigation of conceptual structures and processes. American Psychological Association; 2004. pp. 495–522. 10.1037/10227-018

[23] Zwaan RA. The Immersed Experiencer: Toward An Embodied Theory Of Language Comprehension. Psychol Learn Motiv—Adv Res Theory. 2004;44: 35–62. 10.1016/S0079-7421(03)44002-4

[24] Pulvermüller F, Shtyrov Y, Ilmoniemi R. Brain signatures of meaning access in action word recognition. J Cogn Neurosci. 2005;17: 884–892. 10.1162/0898929054021111 15969907

[25] Barsalou LW, Wiemer-Hastings K. Situating abstract concepts. Grounding Cognition: The Role of Perception and Action in Memory, Language, and Thinking. Cambridge University Press; 2005. pp. 129–163. 10.1017/CBO9780511499968.007

[26] Borghi AM, Barca L, Binkofski F, Castelfranchi C, Pezzulo G, Tummolini L. Words as social tools: Language, sociality and inner grounding in abstract concepts. Phys Life Rev. 2018;29: 120–153. 10.1016/j.plrev.2018.12.001 30573377

[27] Kousta ST, Vigliocco G, Vinson DP, Andrews M, Del Campo E. The Representation of Abstract Words: Why Emotion Matters. J Exp Psychol Gen. 2011;140: 14–34. 10.1037/a0021446 21171803

[28] Ferretti TR, McRae K, Hatherell A. Integrating Verbs, Situation Schemas, and Thematic Role Concepts. J Mem Lang. 2001;44: 516–547. 10.1006/jmla.2000.2728

[29] Barsalou LW. Perceptions of perceptual symbols. Behavioral and Brain Sciences. 1999. pp. 637–660. 10.1017/S0140525X99532147 11301525

[30] Barsalou LW. Situated simulation in the human conceptual system. Language and Cognitive Processes. Psychology Press Ltd; 2003. pp. 513–562. 10.1080/01690960344000026

[31] Wiemer-Hastings K, Xu X. Content differences for abstract and concrete concepts. Cogn Sci. 2005;29: 719–736. 10.1207/s15516709cog0000_33 21702791

[32] Wilson-Mendenhall CD, Simmons WK, Martin A, Barsalou LW. Contextual processing of abstract concepts reveals neural representations of nonlinguistic semantic content. J Cogn Neurosci. 2013;25: 920–935. 10.1162/jocn_a_00361 23363408 PMC3947606

[33] Harpaintner M, Sim EJ, Trumpp NM, Ulrich M, Kiefer M. The grounding of abstract concepts in the motor and visual system: An fMRI study. Cortex. 2020;124: 1–22. 10.1016/j.cortex.2019.10.014 31821905

[34] Dove G. Beyond perceptual symbols: A call for representational pluralism. Cognition. 2009;110: 412–431. 10.1016/j.cognition.2008.11.016 19135654

[35] Dove G. On the need for Embodied and Dis-Embodied Cognition. Front Psychol. 2011;1: 242. 10.3389/fpsyg.2010.00242 21833295 PMC3153846

[36] Dove G. Thinking in Words: Language as an Embodied Medium of Thought. Top Cogn Sci. 2014;6: 371–389. 10.1111/tops.12102 24943737

[37] Pecher D. Curb Your Embodiment. Top Cogn Sci. 2018;10: 501–517. 10.1111/tops.12311 29214726

[38] McRae K, Nedjadrasul D, Pau R, Lo BPH, King L. Abstract Concepts and Pictures of Real-World Situations Activate One Another. Top Cogn Sci. 2018;10: 518–532. 10.1111/tops.12328 29498490

[39] Kuipers JR, Jones MW, Thierry G. Abstract images and words can convey the same meaning. Sci Rep. 2018;8: 1–6. 10.1038/s41598-017-17765-5 29740010 PMC5940816

[40] McRae K, Nedjadrasul D, Pau R, Lo BP-H, King L. Abstract Concepts and Pictures of Real-World Situations Activate One Another. Top Cogn Sci. 2018 [cited 19 Mar 2018]. 10.1111/tops.12328 29498490

[41] Lee MD, Vanpaemel W. Determining informative priors for cognitive models. Psychon Bull Rev. 2018;25: 114–127. 10.3758/s13423-017-1238-3 28194721

[42] JASP—A Fresh Way to Do Statistics. [cited 20 Oct 2020]. Available: https://jasp-stats.org/.

[43] Gronau QF, Ly A, Wagenmakers E-J. Informed Bayesian t -Tests. Am Stat. 2019; 1–14. 10.1080/00031305.2018.1562983

[44] Ly A, Verhagen J, Wagenmakers EJ. Harold Jeffreys’s default Bayes factor hypothesis tests: Explanation, extension, and application in psychology. J Math Psychol. 2016;72: 19–32. 10.1016/j.jmp.2015.06.004

[45] RStudio | Open source & professional software for data science teams—RStudio. [cited 20 Oct 2020]. Available: https://rstudio.com/.

[46] Verhagen J, Wagenmakers E-J. Bayesian Tests to Quantify the Result of a Replication Attempt. 2014 [cited 10 Mar 2020]. 10.1037/a0036731 24867486

[47] Bayarri MJ, Mayoral AM. Bayesian Design of “Successful” Replications. The American Statistician. Taylor & Francis, Ltd.American Statistical Association; pp. 207–214. 10.1198/000313001317098185

[48] Rouder JN, Morey RD. A Bayes factor meta-analysis of Bem’s ESP claim. Psychon Bull Rev. 2011;18: 682–689. 10.3758/s13423-011-0088-7 21573926

[49] Kruschke JK. Bayesian data analysis. Wiley Interdiscip Rev Cogn Sci. 2010;1: 658–676. 10.1002/wcs.72 26271651

[50] Liu CC, Aitkin M. Bayes factors: Prior sensitivity and model generalizability. J Math Psychol. 2008;52: 362–375. 10.1016/j.jmp.2008.03.002

[51] Vanpaemel W. Prior sensitivity in theory testing: An apologia for the Bayes factor. J Math Psychol. 2010;54: 491–498. 10.1016/j.jmp.2010.07.003

[52] Mathôt S. Mathôt S. (2017). Bayes Like a Baws: Interpreting Bayesian Repeated Measures in JASP. [Google Scholar]—Recherche Google. [cited 20 Oct 2020]. Available: https://www.google.com/search?q=Mathôt+S.+(2017).+Bayes+Like+a+Baws%3A+Interpreting+Bayesian+Repeated+Measures+in+JASP.+%5BGoogle+Scholar%5D&ie=utf-8&oe=utf-8&aq=t.

[53] Recchia G, Jones MN. The semantic richness of abstract concepts. Front Hum Neurosci. 2012;6. 10.3389/fnhum.2012.00315 23205008 PMC3506984

[54] Smith EE, Shoben EJ, Rips LJ. Structure and process in semantic memory: A featural model for semantic decisions. Psychol Rev. 1974;81: 214–241. 10.1037/h0036351

[55] Bolognesi M, Vernillo P. How abstract concepts emerge from metaphorical images: The metonymic way. Lang Commun. 2019;69: 26–41. 10.1016/j.langcom.2019.05.003

