# Supplementary material for: Intangible features extraction in the processing of abstract concepts: Evidence from picture-word priming
Source: PLoS One. 2021 May 11;16(5):e0251448. doi: 10.1371/journal.pone.0251448 (PMC8112679; doi:10.1371/journal.pone.0251448)
Supplement: S1 Appendix — (DOCX) [file pone.0251448.s001.docx]

**Appendices: supplementary analyses**

**Table A.1**. Results of the Bayesian Replication Test.

|  | BF_r0_ | Equality B_01_ | Meta B_10_ |
| --- | --- | --- | --- |
| Original | *N/A* | *N/A* | 30.3 |
| Replication | 12.5 | 5.0 |  |

Note. “Rep B_r0_” is the new Bayes factor test for replication; “Equality B_01_” is the equality-of-effect-size Bayes factor test; and “Meta B_10_” is the fixed-effect meta-analysis Bayes factor test.


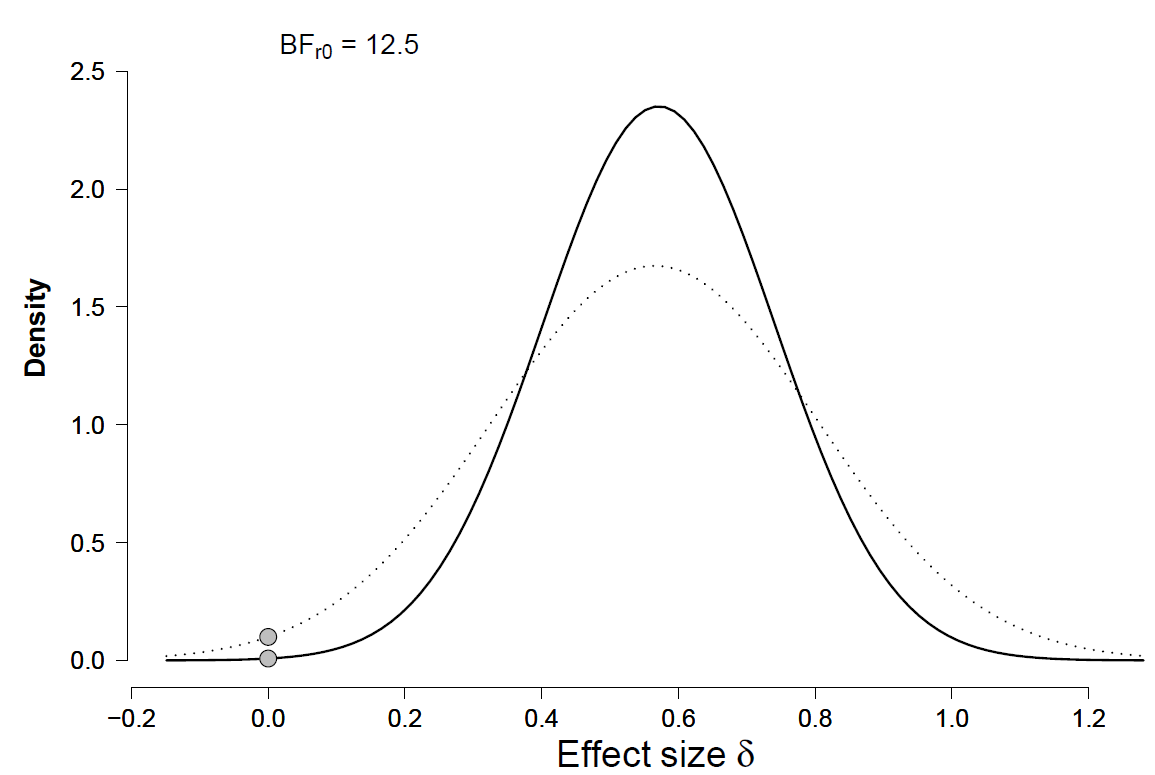


**Figure A.2.** Results of the Bayes factor replication test applied to our replication attempt based on Kuipers et al. (2018).

Note. The dotted line represents the posterior from the original study, which was used as the prior for the effect sizes in the replication tests. The solid line represents the posterior distributions once data from the present replication attempt have been taken into account. The grey dots indicate the ordinates of this prior and posterior for the null hypothesis according to which the effect size is zero. The ratio between these two ordinates gives the result of the replication test [46].
